# Supplementary material for: Versatile Reactivity of MnII Complexes in Reactions with N-Donor Heterocycles: Metamorphosis of Labile Homometallic Pivalates vs. Assembling of Endurable Heterometallic Acetates
Source: Molecules. 2021 Feb 15;26(4):1021. doi: 10.3390/molecules26041021 (PMC7919295; doi:10.3390/molecules26041021)
Supplement: Supplementary file 1 [file molecules-26-01021-s001.pdf]

*Supporting Information*

# Versatile Reactivity of $Mn^{II}$ Complexes in Reactions with N-donor Heterocycles: Metamorphosis of Labile Homometallic Pivalates *vs*. Assembling of Endurable Heterometallic Acetates

**Ruslan A. Polunin [1,†], Igor S. Evstifeev [2], Olivier Cador [3,*], Stéphane Golhen [3], Konstantin S. Gavrilenko [4,5], Anton S. Lytvynenko [1], Nikolay N. Efimov [2], Vadim V. Minin [2], Artem S. Bogomyakov [6], Lahcène Ouahab [3], Sergey V. Kolotilov [1,*], Mikhail A. Kiskin [2,*] and Igor L. Eremenko [2]**

[1] L. V. Pisarzhevskii Institute of Physical Chemistry of the National Academy of Sciences of Ukraine, Prospekt Nauki 31, 03028 Kiev, Ukraine; anton.s.lytvynenko@gmail.com

[2] N. S. Kurnakov Institute of General and Inorganic Chemistry, Russian Academy of Sciences, Leninsky Prosp. 31, 119991 Moscow, GSP-1, Russia; i.evstifeev@gmail.com (I.S.E.); nnefimov@yandex.ru (N.N.E.); minin@igic.ras.ru (V.V.M.); ilerem@igic.ras.ru (I.L.E.)

[3] University of Rennes, CNRS, Institut des Sciences Chimiques de Rennes (ISCR)–UMR 6226, F-35000 Rennes, France; stephane.golhen@univ-rennes1.fr (S.G.); lahcene.ouahab@univ-rennes1.fr (L.O.)

[4] Research-And-Education ChemBioCenter, National Taras Shevchenko University of Kyiv, Chervonotkatska str., 61, 03022 Kiev, Ukraine; kgavrio@gmail.com

[5] Enamine Ltd. 78 Chervonotkatska str., 02660 Kiev, Ukraine

[6] International Tomography Center, Siberia Branch of Russian Academy of Science, Institutskaya str. 3a, 630090 Novosibirsk, Russia; bus@tomo.nsc.ru

* Correspondence: olivier.cador@univ-rennes1.fr (O.C.); s.v.kolotilov@gmail.com (S.V.K.); mkiskin@igic.ras.ru (M.A.K.)

† Deceased.

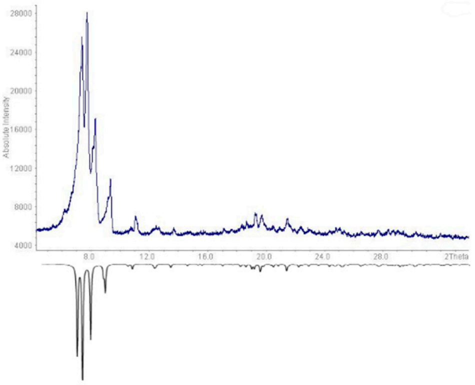


**Figure S1.** Powder X-ray diffraction pattern for **6** (top) along with diffraction calculated from single crystal XRD data (bottom).

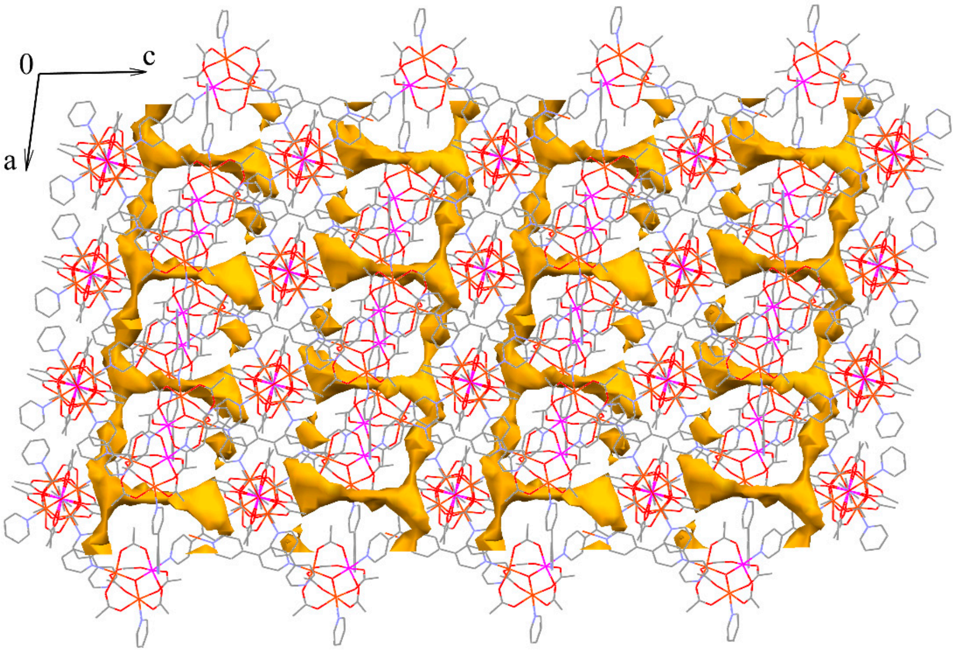


**Figure S2.** Visualization of solvent-accessible voids for **9** using probe molecule with r = 1.4 Å.
